# Associations between Biomarkers of Complement Activation, Galactose-Deficient IgA1 Antibody and the Updated Oxford Pathology Classification of IgA Nephropathy

**DOI:** 10.3390/jcm11144231

**Published:** 2022-07-21

**Authors:** Yun-Ting Juan, Wen-Chih Chiang, Wei-Chou Lin, Cheng-Wen Yang, San-Fang Chou, Ruo-Wei Hung, Yen-Ling Chiu

**Affiliations:** 1 Department of Nephrology, Far Eastern Memorial Hospital, New Taipei City 220, Taiwan; winniesoft7954@gmail.com (Y.-T.J.); candy_1125@hotmail.com (C.-W.Y.); rabit1989114@gmail.com (R.-W.H.); 2 Division of Nephrology, Department of Medicine, National Taiwan University Hospital, Taipei 100, Taiwan; wcchiang@ntu.edu.tw; 3 Department of Pathology, National Taiwan University Hospital, Taipei 100, Taiwan; weichou8@ms52.hinet.net; 4 Department of Medical Research, Far Eastern Memorial Hospital, New Taipei City 220, Taiwan; sfchou1971@gmail.com; 5 Graduate Institute of Medicine and Graduate Program in Biomedical Informatics, Yuan Ze University, Taoyuan 320, Taiwan; 6 Graduate Institute of Clinical Medicine, National Taiwan University College of Medicine, Taipei 100, Taiwan

**Keywords:** complement C5a, factor Ba, galactose-deficient IgA1, IgA nephropathy, Oxford classification

## Abstract

Our prior study indicates a close relationship between alternative complement pathway activation, galactose-deficient IgA1 (Gd-IgA1) concentration and clinical severity of IgA nephropathy (IgAN). Nonetheless, the relationship between complement factors and the updated Oxford classification of IgAN remains unclear. This study enrolled eighty-four previously untreated, biopsy-diagnosed IgAN patients. The clinical and laboratory findings were collected at the time of biopsy. Plasma levels of complement factor C5a, factor Ba and Gd-IgA1 were measured and analyzed. It was found that the levels of proteinuria positively correlated with the updated Oxford classification of mesangial hypercellularity (M), endocapillary hypercellularity (E), tubular atrophy/interstitial fibrosis (T) and crescents (C). In addition, plasma Gd-IgA1 titer was significantly elevated in IgAN patients with tubular atrophy/interstitial fibrosis (T). In separate multivariable logistic regression models, both Gd-IgA1 and factor Ba independently predict higher T scores. The results indicate that both the levels of Gd-IgA1 antibody and biomarkers of the alternative complement pathway activation reflect the Oxford classification of IgAN. Whether these biomarkers can be used to guide therapeutic decisions requires further study.

## 1. Introduction

IgA nephropathy (IgAN) is the most prevalent glomerulonephritis globally [1]. The clinical course and prognosis of IgAN are variant, in which a third of these patients progress to end-stage renal disease [2,3]. The current diagnostic method for IgAN is renal biopsy. Since 2009, the Oxford classification became widely adopted as a popular pathological classification system of the IgAN, which could predict disease activity and prognosis [2,4]. In 2016, an updated Oxford classification, including the MEST-C score, was aimed to further enhance the histopathologic results’ predictive power [5,6,7]. However, renal biopsy is not always performed in actual clinical practice due to the concerns of possible complications [8,9], and it is also rarely performed during clinical follow-ups. 

The development of non-invasive biomarkers is needed to diagnose and evaluate the activity and outcomes of IgAN. In recent years, several studies have reported the role of plasma Gd-IgA1 in IgAN [10,11,12,13]. Some studies have demonstrated the role of complement activation in the pathogenesis of IgAN [14,15,16]. The plasma C3 and factor H levels were reported to be the potential biomarkers associated with IgAN patients [14,16]. Until recently, few studies have examined the complement factor Ba and C5a levels in IgAN patients. Our prior study [17] disclosed the close relationship between complement pathway activation (based on levels of Ba and C5a), Gd-IgA1 concentration and clinical severity of IgAN. 

In this study, we further investigated if the blood levels of factor Ba/C5a and plasma Gd-IgA1 concentration were correlated with histopathological parameters in newly diagnosed IgAN patients. 

## 2. Materials and Methods 

### 2.1. Patients and Blood Sample Collection 

Patients aged between 20~80 years old with the pathologic diagnosis of IgAN were enrolled in this study at the time of renal biopsy between January 2015 and December 2019, as described previously [17]. For the purpose of this study, we only selected patients with renal biopsy reports which were scored according to the updated Oxford classification of IgAN. Renal biopsies were performed in two medical centers: National Taiwan University Hospital and Far Eastern Memorial Hospital. At the time of biopsy, peripheral blood was collected from patients and processed immediately for storage at −80 °C until measurement for biomarkers. This study was approved by the ethics committee of both National Taiwan University Hospital and Far Eastern Memorial Hospital, which was conducted in compliance with the Declaration of Helsinki. Informed consent was obtained from all participants or their legal representatives.

### 2.2. Clinical Parameters

Demographic characteristics, including age, sex, past medical history of diabetes or hypertension and use of angiotensin-converting enzyme inhibitor/angiotensin II receptor blocker (ACEi/ARB), were recorded. The serum levels of creatinine and estimated glomerular filtration rate (eGFR, calculated using the Chronic Kidney Disease Epidemiology Collaboration equations formula for Taiwanese adults) were recorded. The amount of proteinuria was collected in spot urine (urine protein to creatinine ratio, UPCR). 

### 2.3. Measurement of Plasma Gd-IgA1, C5a and Factor Ba 

The plasma Gd-IgA1 levels were measured with enzyme-linked immunosorbent assay (ELISA) (Immuno-Biological Laboratories, Minneapolis, MN, USA). Plasma levels of C5a were measured with the C5a ELISA kit (Aviva Systems Biology, San Diego, CA, USA). Plasma levels of Ba were measured with the MicroVue Ba fragment EIA kit (Quidel Corporation, San Diego, CA, USA). All assays were performed according to the protocols provided by the manufacturers at National Taiwan University Hospital and Far Eastern Memorial Hospital.

### 2.4. Pathology Results

All of the biopsies of IgAN were evaluated by pathologists in two medical centers and applied to the updated Oxford classification. The lesions comprised mesangial hypercellularity (M0 or M1), segmental glomerulosclerosis (S0 or S1), endocapillary hypercellularity (E0 or E1), tubular atrophy/interstitial fibrosis (T0, T1, or T2) and crescents (C0, C1 or C2). 

### 2.5. Statistical Analysis

For baseline characteristics, continuous variables with normal distribution were presented as mean ± standard deviation (SD) and the data not normally distributed were expressed as median (interquartile range). The categorical data were reported as the frequency with percentage. Comparisons of continuous variables in two groups were performed by independent t-test or Mann–Whitney U test as appropriated in data with or without normal distribution. Relationships between two continuous variables with normal distribution were analyzed using Pearson correlation. Multivariable logistic regression analysis was performed for the predictive value of biomarkers on tubular atrophy/interstitial fibrosis. A *p*-value less than 0.05 was considered statistically significant. Statistical analyses were performed with SPSS Statistics Version 26 (IBM) and Prism software, version 9.3.1 (GraphPad Software, San Diego, CA, USA). 

## 3. Results 

### 3.1. Baseline Characteristics 

We included 84 patients with primary IgAN in this study. The mean age of the patients was 44.78 ± 14.02, and 46.4% were males. The average serum creatinine level was 1.61 ± 1.12 mg/dL with an average eGFR of 54.92 ± 33.06 mL/min/1.73 m^2^. Most of the patients had significant proteinuria with an average UPCR of 2.81 ± 3.14 g/g. These clinical parameters indicated that the included patients already had significant renal damage. The median plasma Gd-IgA1 concentration among IgAN patients was 7740 (5221–11826) ng/mL. The median plasma factor Ba and C5a levels in IgAN patients were 2992 (1640–4588) ng/mL and 3617 (1447–4961), respectively. Based on the updated Oxford classification of IgAN, 69 (82%) patients had mesangial hypercellularity (M1), 27 (32%) patients had endocapillary hypercellularity (E1), 77 (92%) patients had segmental glomerulosclerosis (S1), 28 (33%) patients had tubular atrophy/interstitial fibrosis (T1-22%, T2-11%) and 10 (12%) patients had crescents (C1-12%, C2-0%). The baseline characteristics are presented in Table 1. 

### 3.2. Associations of Plasma Biomarkers Level with Clinical Parameters

The Gd-IgA1 level showed negative correlation with eGFR and positive correlation with the UPCR level (r = −0.310, *p* = 0.004 and r = 0.222, *p* = 0.042, respectively, Table A1). The Gd-IgA1 level was positively correlated with the level of factor Ba and C5a (r = 0.240, *p* = 0.028 and r = 0.233, *p* = 0.033, respectively, Table A1). 

The level of plasma factor Ba was negatively correlated with eGFR (r = −0.294, *p* = 0.007, Table A2), and positively correlated with the UPCR level (r = 0.327, *p* = 0.002, Table A2). The factor Ba level also had positive association with the C5a level (r = 0.507, *p* < 0.0001, Table A2). However, the level of plasma C5a had no correlation with eGFR and proteinuria (Table A3). These results suggested that renal function and the UPCR level were associated with the plasma levels of Gd-IgA1 and factor Ba but not with C5a. In summary, these findings were consistent with our prior study [17]. 

### 3.3. Associations of Pathological Parameters with Clinical Parameters

**Figure 1:**
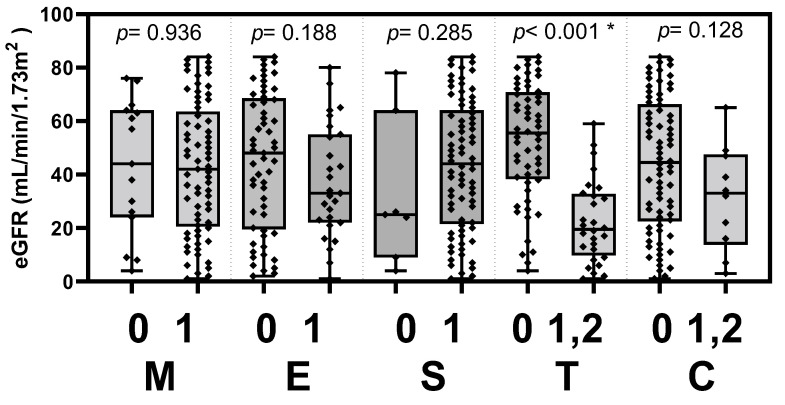
eGFR in the Oxford classification MEST-C score-stratified groups. Levels of eGFR were significantly decreased in IgAN patients with tubular atrophy/interstitial fibrosis (*p* < 0.001). eGFR, estimated glomerular filtration rate; M, mesangial hypercellularity; E, endocapillary hypercellularity; S, segmental glomerulosclerosis; T, tubular atrophy/interstitial fibrosis; C, cellular or fibrocellular crescents. * *p* < 0.05.

**Figure 2:**
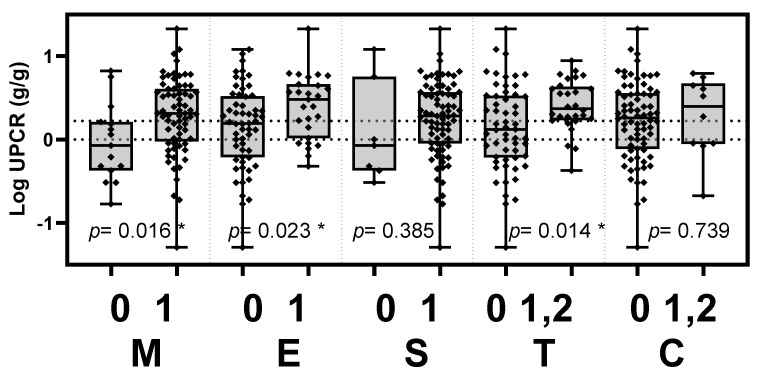
Levels of proteinuria in the Oxford classification MEST-C score-stratified groups. UPCR levels were significantly elevated in IgAN patients with mesangial hypercellularity (*p* = 0.016), endocapillary hypercellularity (*p* = 0.023) and tubular atrophy/interstitial fibrosis (*p* = 0.014). UPCR, urinary protein to creatinine ratio; M, mesangial hypercellularity; E, endocapillary hypercellularity; S, segmental glomerulosclerosis; T, tubular atrophy/interstitial fibrosis; C, cellular or fibrocellular crescents. * *p* < 0.05.

The clinical activities of the UPCR level and eGFR were tested to determine if they associated with the histopathological score according to the updated Oxford classification. The patients of IgAN were stratified into two groups based on the updated Oxford classification with or without the histopathology of mesangial hypercellularity (M), endocapillary hypercellularity (E), segmental glomerulosclerosis (S), tubular atrophy/interstitial fibrosis (T) or crescents (C). The UPCR levels were significantly elevated in patients with mesangial hypercellularity, endocapillary hypercellularity and tubular atrophy/interstitial fibrosis (*p* = 0.016, 0.023 and 0.014, respectively). The eGFR was significantly decreased in patients with tubular atrophy/interstitial fibrosis (*p* < 0.001). The differences in clinical parameters between MEST-C score-stratified groups are presented in Table 2, Figure 1 and Figure 2. 

### 3.4. Associations between Plasma Biomarker Levels with Pathological Parameters 

The titers of Gd-IgA1, factor Ba and C5a in the Oxford classification MEST-C score-stratified groups were summarized in Table 3. 

**Figure 3:**
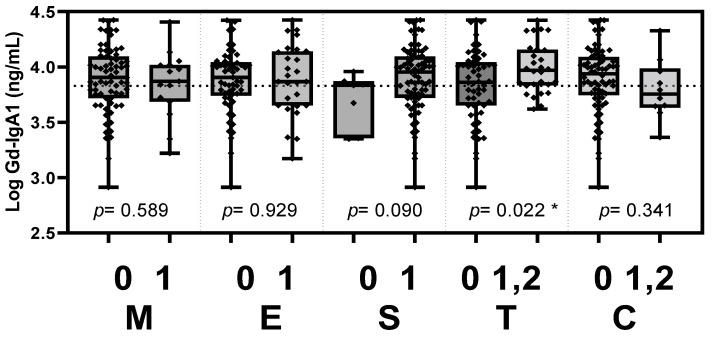
Plasma Gd-IgA1 levels stratified according to the Oxford MEST-C criteria. Plasma Gd-IgA1 level was significantly elevated in patients with tubular atrophy/interstitial fibrosis (*p* = 0.022). Gd-IgA1, galactose-deficient IgA1; M, mesangial hypercellularity; E, endocapillary hypercellularity; S, segmental glomerulosclerosis; T, tubular atrophy/interstitial fibrosis; C, cellular or fibrocellular crescents. * *p* < 0.05.

**Figure 4:**
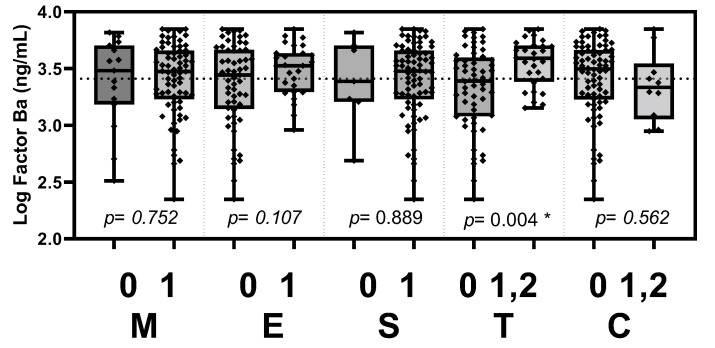
Plasma factor Ba levels stratified according to the Oxford MEST-C criteria. Plasma factor Ba level was significantly elevated in patients with tubular atrophy/interstitial fibrosis (*p* = 0.004). M, mesangial hypercellularity; E, endocapillary hypercellularity; S, segmental glomerulosclerosis; T, tubular atrophy/interstitial fibrosis; C, cellular or fibrocellular crescents. * *p* < 0.05.

**Figure 5:**
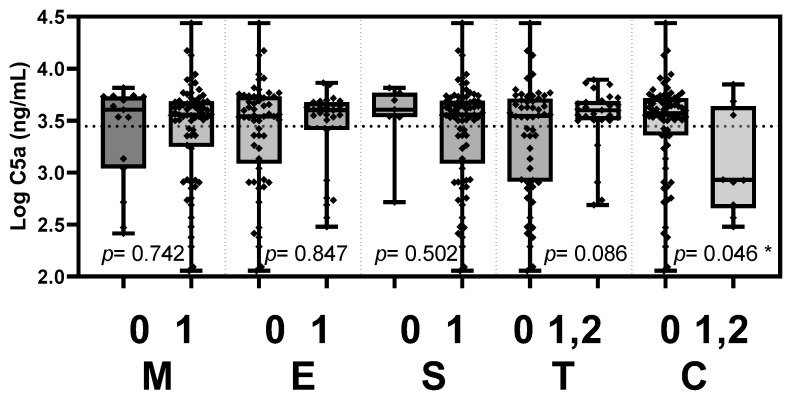
Plasma C5a levels stratified according to the Oxford MEST-C criteria. Plasma C5a level was elevated in patients with crescents (*p* = 0.046). M, mesangial hypercellularity; E, endocapillary hypercellularity; S, segmental glomerulosclerosis; T, tubular atrophy/interstitial fibrosis; C, cellular or fibrocellular crescents. * *p* < 0.05.

Based on the updated Oxford classification, the plasma Gd-IgA1 level was significantly elevated in patients with tubular atrophy/interstitial fibrosis (*p* = 0.022, Figure 3). Plasma Ba level was significantly elevated in patients with tubular atrophy/interstitial fibrosis (*p* = 0.004, Figure 4). Plasma C5a level was elevated in patients with crescents (*p* = 0.046, Figure 5). 

### 3.5. Multivariable Logistical Regression Analyses of Tubular Atrophy/Interstitial Fibrosis

As shown in Table 4, two models of multivariate logistic regression analyses both revealed that a higher factor Ba level was independently associated with the Oxford T scores after being adjusted according to age, gender and other risk factors including diabetes, UPCR, and usage of ACEI/ARB. 

Plasma Gd-IgA1 level was also associated with the Oxford T scores in multivariable model 1, which was adjusted according to age and gender (*p* = 0.037). However, the association was not significant between the plasma Gd-IgA1 level and the Oxford T scores in model 2 (*p* = 0.082, Table 5).

## 4. Discussion 

In this study, we found that the (1) plasma biomarkers of Gd-IgA1 and factor Ba level were both negatively correlated with eGFR and positively correlated with UPCR in IgAN patients. (2) The UPCR level was significantly elevated in IgAN patients with mesangial hypercellularity (M), endocapillary hypercellularity (E) and tubular atrophy/interstitial fibrosis (T) according to the updated Oxford classification. Nevertheless, the eGFR level had a significant association with T score only. (3) The plasma biomarkers of Gd-IgA1 and factor Ba level were significantly elevated in IgAN patients with tubular atrophy/interstitial fibrosis (T). Plasma C5a level was significantly elevated in IgAN patients with crescents (C). 

Previous studies had demonstrated the correlation between plasma Gd-IgA1 and clinical parameters in IgAN patients. Kim et al. [10] demonstrated that the plasma level of Gd-IgA1 was negatively correlated with eGFR and positively correlated with the frequency of CKD progression. One study reported the trend of lower eGFR and higher proteinuria with increasing Gd-IgA1 [18]. In contrast, other studies reported that there was no correlation between the plasma Gd-IgA1 level and parameters of disease severity, including eGFR and UPCR [11,19]. Other studies reported that the levels of Gd-IgA1 were associated with disease progression, even though there was no significant correlation between plasma Gd-IgA1 levels and clinical findings at the time of IgAN diagnosis [18,20,21]. The discrepancy in the relationship between the Gd-IgA1 level and disease activity may be related to the baseline disease severity of the enrolled IgAN patients. Gd-IgA1 levels may have a stronger clinical impact in moderate to severe IgAN patients than in patients with mild disease activity [17]. In this current study, not only was the Gd-IgA1 level positively correlated with disease activity, but the alternative complement of the plasma factor Ba level was also correlated with clinical parameters. 

The relationship between plasma Gd-IgA1 concentrations and pathological results has already been described in several studies [10,13,18,22]. The Gd-IgA1 level was positively correlated with advanced pathological findings and future renal function decline [13]. Kim et al. [10] demonstrated that the plasma Gd-IgA1 level was associated with tubular atrophy/interstitial fibrosis in IgAN patients. Two recent studies [13,18] both reported that the plasma Gd-IgA1 level was significantly higher in IgAN patients with segmental glomerulosclerosis and tubular atrophy/interstitial fibrosis. Consistent with the above studies, we observed that the plasma Gd-IgA1 level was significantly elevated in IgAN patients with tubular atrophy/interstitial fibrosis. In addition, the plasma Gd-IgA1 level was positively associated with tubular atrophy/interstitial fibrosis in IgAN patients after being adjusted for age and gender. Nguyen et al. [18] were the first to show that higher plasma Gd-IgA1 titers were associated with stronger mesangial cell inflammatory response with production of a greater amount of monocyte chemoattractant protein-1 (MCP-1) in vitro, which in turn was associated with severe histologic changes in S and T scores according to the updated Oxford classifications. To the best of our knowledge, this study is the first to demonstrate that the plasma factor Ba and C5a levels were significantly elevated in IgAN patients with tubular atrophy/interstitial fibrosis (T) and crescents (C), respectively. In our study, we confirmed the significant association between plasma factor Ba levels and higher grade of tubular atrophy/interstitial fibrosis in IgAN patients, after being adjusted for age, gender, diabetes, UPCR and usage of ACEI/ARB. Such findings advocate for the causal role of the alternative pathway in the pathogenesis of IgAN. Since some early results from clinical trials targeting complement pathways have been showing promise in IgAN [23], the levels of complement pathway activation products thus should be investigated for their role in predicting disease outcomes and guiding treatment decisions.

Renal biopsy is the current gold standard for diagnosis and assessment of IgAN. Pathology diagnosis and staging provide valuable information on disease severity and prognosis. Nevertheless, patients may refuse renal biopsy and it might not be available in some instances. Although proteinuria is widely known to be associated with the prognosis of patients with various types of glomerulonephritis, it is challenging to determine acute glomerular disease or chronic lesions simply by the degree of proteinuria. Thus, a reliable blood-based IgAN biomarker may be of clinical significance. In addition, by combining pathology, clinical parameters and blood-based biomarkers, IgAN patients may benefit from a new disease prediction algorithm to guide disease management in the future. Such an approach has been shown by Barbour et al. [24], who had presented the accuracy and validation of a new international risk-prediction tool in IgAN.

Our study had several limitations. First, the plasma biomarker level of Gd-IgA1, factor Ba and C5a were only measured once in each patient. Levels of Gd-IgA1 and other biomarkers vary a lot between individual patients and thus the interpretation of this biomarkers for each patient should be carried out with caution. Second, the UPCR level was determined by spot urine, but not 24 h urine. Longitudinal analysis and more detailed clinical information may further solidify our findings. The findings should also be validated in additional studies with an adequate sample size.

## 5. Conclusions

Our study is the first to establish the relationship between factor Ba/C5a and the Oxford pathology scores. These findings warrant additional studies to test their potential clinical applications.

## Figures and Tables

**Table 1:** Baseline characteristics of all IgAN patients.

	Total (*n* = 84)
Age (years)	44.78 ± 14.02
Male (%)	39 (46.4%)
Diabetes (%)	7 (8.33%)
Hypertension (%)	38 (45.8%)
Use of ACEI/ARB (%)	53 (63.1%)
UPCR (g/g)	2.81 ± 3.14
Creatinine (mg/dL)	1.61 ± 1.12
eGFR (mL/min/1.73 m^2^)	54.92 ± 33.06
Gd-IgA1 Ab titer (ng/mL)	7740 (5221–11,826)
Factor C5a (ng/mL)	3617 (1447–4961)
Factor Ba (ng/mL)	2992 (1640–4588)
Oxford Classification	*n* (%)
M0	15 (18%)
M1	69 (82%)
E0	57 (68%)
E1	27 (32%)
S0	7 (8%)
S1	77 (92%)
T0	56 (67%)
T1	19 (22%)
T2	9 (11%)
C0	74 (88%)
C1	10 (12%)
C2	0 (0%)

Normally distributed continuous variables including age, UPCR, creatinine and eGFR are expressed as mean ± SD. Non-normally distributed continuous variables such as Gd-IgA1 Ab titer, C5a and factor Ba level are expressed as median (interquartile range). ACEi/ARB, angiotensin-converting enzyme inhibitor/angiotensin II receptor blocker; UPCR, urine protein to creatinine ratio; eGFR, estimated glomerular filtration rate; M, mesangial hypercellularity; E, endocapillary hypercellularity; S, segmental glomerulosclerosis; T, tubular atrophy/interstitial fibrosis; C, crescents; Gd-IgA1, galactose-deficient IgA1.

**Table 2:** The clinical parameters in the Oxford classification MEST-C score-stratified groups.

Oxford Classification	Score	Log UPCR (g/g)	*p*	eGFR (mL/min/1.73 m^2^)	*p*
M	0	−0.035 ± 0.463	0.016 *	59.72 ± 35.56	0.936
	1	0.287 ± 0.457		61.67 ± 41.08	
E	0	0.149 ± 0.490	0.023 *	66.02 ± 42.92	0.188
	1	0.398 ± 0.386		51.38 ± 31.27	
S	0	0.080 ± 0.606	0.385	50.49 ± 45.99	0.285
	1	0.243 ± 0.460		62.30 ± 39.56	
T	0	0.140 ± 0.515	0.014 *	76.72 ± 38.98	<0.001 *
	1, 2	0.407 ± 0.308		30.52 ± 18.36	
C	0	0.223 ± 0.4742	0.739	63.90 ± 40.94	0.128
	1, 2	0.277 ± 0.475		42.18 ± 25.87	

Log UPCR and eGFR were presented as the mean ± SD. The mean level in score 0 was compared to those in score 1 or score 1, 2 (by Oxford MEST-C classification) and was analyzed using independent t-test. * *p* < 0.05. UPCR, urine protein to creatinine ratio; eGFR, estimated glomerular filtration rate; M, mesangial hypercellularity; E, endocapillary hypercellularity; S, segmental glomerulosclerosis; T, tubular atrophy/interstitial fibrosis; C, crescents.

**Table 3:** Comparisons of biomarker levels between the groups stratified according to the Oxford classification of MEST-C histopathology.

Oxford Classification	Score	Log Gd-IgA1 (ng/mL)	*p*	Log C5a (ng/mL)	*p*	Log Ba (ng/mL)	*p*
M	0	3.845 ± 0.303	0.589	3.369 ± 0.488	0.742	3.378 ± 0.394	0.752
	1	3.891 ± 0.303		3.415 ± 0.487		3.408 ± 0.322	
E	0	3.881 ± 0.295	0.929	3.400 ± 0.518	0.847	3.362 ± 0.369	0.107
	1	3.887 ± 0.320		3.422 ± 0.414		3.488 ± 0.224	
S	0	3.698 ± 0.250	0.090	3.525 ± 0.377	0.502	3.386 ± 0.388	0.889
	1	3.900 ± 0.302		3.396 ± 0.497		3.404 ± 0.331	
T	0	3.830 ± 0.322	0.022 *	3.343 ± 0.545	0.086	3.330 ± 0.361	0.004 *
	1,2	3.989 ± 0.226		3.535 ± 0.300		3.549 ± 0.207	
C	0	3.895 ± 0.305	0.341	3.445 ± 0.471	0.046 *	3.411 ± 0.338	0.562
	1,2	3.797 ± 0.272		3.121 ± 0.507		3.345 ± 0.305	

Levels of Gd-IgA1, C5a and factor Ba were log-transformed and analyzed by independent t-test. Continuous variables are presented as the mean ± SD. * indicates significant difference between the score-stratified groups (*p* < 0.05). Gd-IgA1, galactose-deficient IgA1; M, mesangial hypercellularity; E, endocapillary hypercellularity; S, segmental glomerulosclerosis; T, tubular atrophy/interstitial fibrosis; C, cellular or fibrocellular crescents.

**Table 4:** Associations between the plasma factor Ba level with tubular atrophy/interstitial fibrosis (the Oxford T scores).

Independent Variables in Model (Dependent Variable: T Scores)	Model 1		Model 2	
	OR (95% CI)	*p* Value	OR (95% CI)	*p* Value
Age	1.006 (0.97–1.04)	0.745	0.997 (0.96–1.04)	0.892
Gender (Male)	0.420 (0.15–1.15)	0.091	0.443 (0.16–1.25)	0.125
Diabetes			1.149 (0.17–7.77)	0.886
Use of ACEI/ARB			0.931 (0.32–2.71)	0.896
Log UPCR (g/g)			3.037 (0.82–11.27)	0.097
Log Factor Ba (ng/mL)	13.18 (1.92–90.45)	0.009 *	9.733 (1.31–72.57)	0.026 *

Multivariate logistic regression analyses: model 1 (age, gender) and model 2 (age, gender, diabetes mellitus, use of ACEI/ARB, UPCR) were used to investigate the association between the plasma factor Ba level and tubular atrophy/interstitial fibrosis in IgAN patients. OR, odds ratio; 95% CI, 95% confidence interval. * *p* < 0.05.

**Table 5:** Associations between the plasma Gd-IgA1 level with tubular atrophy/interstitial fibrosis (the Oxford T scores).

Independent Variables in Model (Dependent Variable: T Scores)	Model 1		Model 2	
	OR (95% CI)	*p* Value	OR (95% CI)	*p* Value
Age	1.004 (0.97–1.04)	0.823	0.995 (0.96–1.04)	0.815
Gender (Male)	0.423 (0.16–1.13)	0.085	0.454 (0.16–1.27)	0.133
Diabetes			1.498 (0.22–10.10)	0.678
Use of ACEI/ARB			0.913 (0.32–2.61)	0.866
Log UPCR (g/g)			3.198 (0.94–10.88)	0.063
Log Gd-IgA1 (ng/mL)	6.501 (1.11–37.92)	0.037 *	5.349 (0.81–35.50)	0.082

Multivariate logistic regression analyses: model 1 (age, gender) and model 2 (age, gender, diabetes mellitus, use of ACEI/ARB, UPCR) were used to investigate the association between the plasma Gd-IgA1 level and tubular atrophy/interstitial fibrosis in IgAN patients. OR, odds ratio; 95% CI, 95% confidence interval. * *p* < 0.05.

## Data Availability

The data presented in this study are available on request from the corresponding author.

## Appendix A

**Table A1:** Correlation analysis of Gd-IgA1 with clinical parameters and the levels of factor Ba and C5a.

Variable	Log Gd-IgA1	
	Correlation Coefficient (r)	*p*
eGFR	−0.310	0.004 *
Log UPCR	0.222	0.042 *
Log factor Ba	0.240	0.028 *
Log factor C5a	0.233	0.033 *

The correlation analysis was performed using Pearson’s correlation test after logarithmic transformation of the levels of Gd-IgA1, UPCR, factor Ba and C5a. *, *p* < 0.05, indicates a significant correlation between the two variables. Gd-IgA1, galactose-deficient IgA1; eGFR, estimated glomerular filtration rate; UPCR, urine protein to creatinine ratio.

**Table A2:** Correlation analysis of the plasma factor Ba level with clinical parameters and plasma factor C5a level.

Variable	Log Factor Ba	
	Correlation Coefficient (r)	*p*
eGFR	−0.294	0.007 *
Log UPCR	0.327	0.002 *
Log factor C5a	0.507	<0.0001 *

The correlation analysis was performed using Pearson’s correlation test after logarithmic transformation of the levels of factor Ba, UPCR and C5a. *, *p* < 0.05, indicates a significant correlation between the two variables. eGFR, estimated glomerular filtration rate; UPCR, urine protein to creatinine ratio.

**Table A3:** Correlation analysis of the plasma factor C5a level with clinical parameters.

Variable	Log C5a	
	Correlation Coefficient (r)	*p*
eGFR	−0.073	0.512
Log UPCR	0.145	0.188

The correlation analysis was performed using Pearson’s correlation test after logarithmic transformation of the levels of C5a and UPCR. eGFR, estimated glomerular filtration rate; UPCR, urine protein to creatinine ratio.
